# The Impact of Atrial Fibrillation Treatment Strategies on Cognitive Function

**DOI:** 10.3390/jcm12093050

**Published:** 2023-04-22

**Authors:** Neil Bodagh, Irum Kotadia, Ali Gharaviri, Fernando Zelaya, Jonathan Birns, Ajay Bhalla, Peter Sommerville, Steven Niederer, Mark O’Neill, Steven E. Williams

**Affiliations:** 1 School of Biomedical Engineering and Imaging Sciences, King’s College London, London SE1 7EH, UK; 2 Centre for Cardiovascular Science, University of Edinburgh, Edinburgh EH16 4TJ, UK; 3 St Thomas’ Hospital, Guys and St Thomas’ NHS Foundation Trust, London SE1 7EH, UK

**Keywords:** atrial fibrillation, dementia, cognitive decline, anticoagulation, rate control, rhythm control

## Abstract

There is increasing evidence to suggest that atrial fibrillation is associated with a heightened risk of dementia. The mechanism of interaction is unclear. Atrial fibrillation-induced cerebral infarcts, hypoperfusion, systemic inflammation, and anticoagulant therapy-induced cerebral microbleeds, have been proposed to explain the link between these conditions. An understanding of the pathogenesis of atrial fibrillation-associated cognitive decline may enable the development of treatment strategies targeted towards the prevention of dementia in atrial fibrillation patients. The aim of this review is to explore the impact that existing atrial fibrillation treatment strategies may have on cognition and the putative mechanisms linking the two conditions. This review examines how components of the ‘Atrial Fibrillation Better Care pathway’ (stroke risk reduction, rhythm control, rate control, and risk factor management) may influence the trajectory of atrial fibrillation-associated cognitive decline. The requirements for further prospective studies to understand the mechanistic link between atrial fibrillation and dementia and to develop treatment strategies targeted towards the prevention of atrial fibrillation-associated cognitive decline, are highlighted.

## 1. Introduction

There is an ever-accumulating evidence base to suggest a link between atrial fibrillation and cognitive decline [1]. The mechanism of interaction between the two disease states is unknown. Both conditions are projected to increase in prevalence in the future [2,3] and are consequently likely to exert a significant impact from economic and public health perspectives.

The clinical complexity of care for patients with atrial fibrillation has resulted in the development of the ‘Atrial Fibrillation Better Care pathway’, which has been designed to provide an integrated, holistic approach to atrial fibrillation management [4]. The pathway emphasises the requirement for stroke risk reduction, symptom management (rhythm and rate control), and co-morbidity optimisation (including lifestyle changes) to optimise treatment for atrial fibrillation patients. Studies have demonstrated that adherence to pathway appears to be associated with a lower risk of adverse outcomes such as mortality, thromboembolism, and major adverse cardiac events [5,6]. Furthermore, a recent prospective, cluster randomised trial demonstrated that the application of a mobile health technology-implemented ‘Atrial Fibrillation Better Care pathway’ was associated with improved outcomes in older atrial fibrillation patients aged ≥75 years old [7]. Interestingly, an observational study has also shown that implementation of the ‘Atrial Fibrillation Better Care Pathway’ may be associated with a reduced risk of dementia [8]. Whilst such a study can only infer association as opposed to causation, it is certainly feasible to speculate that the pathway’s integrated care approach may result in improved cognitive outcomes amongst atrial fibrillation patients.

**Figure 1:**
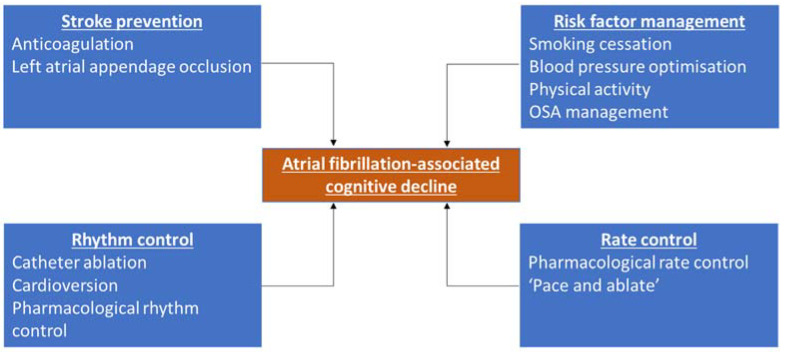
Treatment strategies which may affect atrial fibrillation-associated cognitive decline. OSA = obstructive sleep apnoea.

The aim of this review is to discuss how aspects of the ‘Atrial Fibrillation Better Care pathway’ may potentially modify the risk of atrial fibrillation-associated cognitive decline (Figure 1). An emphasis will be placed on the interaction between atrial fibrillation treatment modalities and the candidate pathophysiological mechanisms linking the two conditions.

## 2. Pathogenesis of Atrial Fibrillation-Associated Cognitive Decline

**Figure 2:**
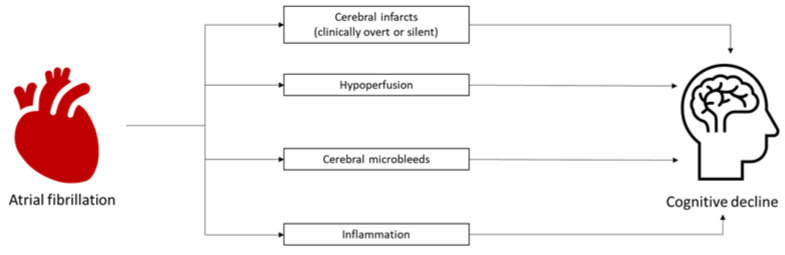
Proposed mechanisms linking atrial fibrillation and cognitive decline.

Several mechanisms have been proposed to explain the association between atrial fibrillation and cognitive decline. Since atrial fibrillation confers an increased risk of stroke [9], it is postulated that atrial fibrillation may be linked with dementia through the increased risk of cerebral infarction. Whilst patients who have a history of stroke and atrial fibrillation do have an increased risk of dementia [10], an increased risk of dementia has also been observed in atrial fibrillation patients without a history of clinical stroke [11]. The presence of shared risk factors (e.g., diabetes mellitus, hypertension) may explain the link between cognitive decline and atrial fibrillation in the absence of clinical stroke. However, studies which control for these risk factors also continue to demonstrate a link [12,13]. These observations have prompted further investigation into the link between atrial fibrillation and cognitive decline. Several mechanisms, including silent cerebral infarcts, microbleeds, hypoperfusion, and inflammation, have also been proposed to explain the relationship between the two disease states (Figure 2) [14,15,16,17].

The observed association between atrial fibrillation and an increased risk of silent cerebral infarcts [18] has resulted in the suggestion that atrial fibrillation-induced silent cerebral infarction may contribute to cognitive decline. Indeed, previous studies have demonstrated that atrial fibrillation patients who have clinically silent, large non-cortical infarcts appear to have lower cognitive function assessment scores compared with those without large non-cortical infarcts [19]. Furthermore, a cohort study of 1227 atrial fibrillation patients identified new cerebral infarcts in 5.5% of patients at 2 years and found that the development of new infarcts was associated with cognitive decline, irrespective of whether infarcts were clinically overt or silent [16]. These studies therefore suggest a high incidence and prevalence of silent cerebral infarcts amongst atrial fibrillation patients and that their presence may be associated with cognitive decline.

Atrial cardiomyopathy, characterised by structural and functional changes in the atria [20], has also been associated with an increased risk of cerebral infarction independent of atrial fibrillation [21]. Consequently, it has been suggested that changes in the structure and function of the left atrium may be independently associated with cognitive impairment. Indeed, a recent observational study has demonstrated that patients with atrial cardiomyopathy (defined using blood biomarkers, electrocardiogram parameters, and echocardiogram measurements) appear to have an increased risk of dementia, independent of atrial fibrillation [22]. Furthermore, it has also been shown that patients with atrial cardiomyopathy have increased quantities of positron emission tomography markers of brain beta-amyloid, a marker of Alzheimer’s disease [23]. Future studies should aim to examine whether there is a causal relationship between atrial cardiomyopathy and dementia and ascertain whether any potential relationship is independent of atrial fibrillation.

Cerebral microbleeds are frequently encountered amongst atrial fibrillation patients, with an estimated prevalence of 28.3%, and their presence has been associated with a higher risk of both thromboembolic and bleeding events [24]. Amongst the general population, cerebral microbleeds have been linked to an increased risk of dementia [15]. These observations have resulted in speculation that cerebral microbleeds may contribute to atrial fibrillation-associated cognitive decline. Amongst atrial fibrillation patients with cerebral microbleeds however, Conen et al., failed to demonstrate a link between their presence and cognitive impairment [19]. Consequently, it is currently unclear whether cerebral microbleeds contribute to atrial fibrillation-associated cognitive decline.

The aetiology of cerebral microbleeds in atrial fibrillation patients is also currently unknown. Anticoagulant therapy, used to mitigate the risk of stroke [25], has been associated with an increased risk of cerebral microbleeds [26] and it has therefore been suggested that anticoagulant therapy may cause cerebral microbleeds and contribute to atrial fibrillation-associated cognitive decline [27]. However, a study by Saito et al., failed to demonstrate an increased risk of cerebral microbleeds in atrial fibrillation patients receiving direct-acting oral anticoagulant therapy, although only over a short one-year follow-up period [28]. Cerebral amyloid angiopathy, characterised by amyloid-beta peptide deposition in small- to medium-sized blood vessels, is known to predispose to intracerebral haemorrhage [29]. Further studies should examine whether the condition contributes to the development of atrial fibrillation-associated microbleeds. Studies which have attempted to examine for a link between cerebral microbleeds and cognitive impairment have predominantly used magnetic resonance imaging, which is unable to ascertain the aetiology of cerebral microbleeds. Future studies should aim to determine the aetiology of cerebral microbleeds in atrial fibrillation patients and examine whether their presence affects cognition.

Atrial fibrillation has additionally been associated with reduced cerebral blood flow [14,30], and it has been postulated that this may lead to atrial fibrillation-associated cognitive decline [27]. In the general population, cerebral hypoperfusion has been associated with a heightened risk of dementia [31]. However, there is currently no direct evidence examining whether atrial fibrillation associated-cerebral hypoperfusion is associated with an increased risk of cognitive decline. Further study is therefore required to investigate whether the observed reduction in cerebral blood flow causes cognitive impairment.

Inflammation may contribute to the pathogenesis of both dementia [32] and atrial fibrillation [33]. Inflammation has consequently been proposed as a candidate pathophysiological mechanism linking the two disease states. In patients with Alzheimer’s disease, studies have demonstrated the accumulation of activated immune cells in human brain tissue [34] and cerebrospinal fluid [35]. It has been proposed that blood-brain barrier disruptions may facilitate the entry of inflammatory mediators into the central nervous system, leading to the pathogenesis of neurodegenerative diseases such as Alzheimer’s disease [36]. Systemic diseases such as hypertension and obesity may trigger the release of inflammatory markers which cause structural and electrical remodelling leading to atrial fibrillation [37]. Atrial fibrillation itself may trigger inflammation [38] and alter cerebral blood flow, leading to perturbed functioning of the blood-brain barrier [39]. It has consequently been hypothesised that atrial fibrillation-induced inflammation and subsequent blood-brain barrier disruption may explain the link between atrial fibrillation and cognitive decline. At present, the evidence for this link is limited. Further studies are required to increase our understanding of the role of inflammation in the individual disease states themselves and to subsequently aim to establish whether inflammation may contribute to atrial fibrillation-associated cognitive decline.

Whilst further studies are required to delineate the pathophysiological mechanisms linking atrial fibrillation and cognitive decline, some of the mechanisms are potentially modifiable using contemporary treatments. Therefore, in the future, treatment strategies may be designed and tailored to modify the progression of atrial fibrillation-associated cognitive decline. In the following sections, candidate interactions between existing treatment modalities and future cognitive impairment are discussed.

## 3. Stroke Risk Reduction

### 3.1. Anticoagulation

Based on the hypothesis that atrial fibrillation-induced cerebral emboli may cause cognitive decline [16], it has been postulated that oral anticoagulation may lower the risk of atrial fibrillation-related dementia. This theory is supported by observational studies which have demonstrated that atrial fibrillation patients taking oral anticoagulation have a lower risk of developing dementia than those not on anticoagulant therapy [40,41]. Nevertheless, it is important to interpret such findings with caution. Unmeasured confounders may have contributed to the results obtained, and it is important to highlight that physicians may have been less likely to prescribe anticoagulant therapy to older patients with worse baseline cognitive function due to a perceived increased bleeding risk. This presents an alternate explanation for the purported reduced risk of dementia associated with anticoagulant therapy.

At present, anticoagulant therapy is initiated according to the CHA_2_DS_2_-VASc score (which includes congestive heart failure, hypertension, age (≥75 years), diabetes, stroke/transient ischaemic attack, vascular disease, age (65–74 years), and sex (female)), which is associated with stroke risk [42]. For patients deemed to be at a lower risk of stroke, anticoagulant treatment is not currently indicated [43]. Friberg et al., investigated whether anticoagulation in atrial fibrillation patients at low established risk of systemic thromboembolism would be of benefit when the risks of dementia and intracranial bleeding were considered [41]. The authors performed a retrospective study of national registries of individuals in Sweden with a hospital diagnosis of atrial fibrillation and found that 43% of patients were using oral anticoagulant therapy (39,160 out of 91,254 patients) despite a low risk of ischaemic stroke. Overall, oral anticoagulant therapy use was associated with a lower risk of dementia (hazard ratio 0.60, 95% confidence interval 0.46–0.77). Whilst such a result is of interest, it is important to balance this against the increased risk of bleeding, particularly in younger patients. Using a composite endpoint of dementia, ischaemic stroke, or intracranial bleeding, the authors found that anticoagulant therapy appeared to be associated with a higher risk of the composite endpoint in patients aged <60 years. In patients aged >65 years, anticoagulant therapy was associated with a net benefit.

The impact of anticoagulant therapy on cognitive outcomes in subclinical atrial fibrillation is currently unknown. A recent sub-study of the LOOP study aimed to investigate the relationship between subclinical atrial fibrillation, anticoagulation, and cognitive function over a three-year follow-up period [44]. The original LOOP study investigated the impact of using implantable loop recorder monitoring to detect atrial fibrillation on outcomes in 70–90-year-old patients with ≥1 stroke risk factor (i.e., hypertension, diabetes, prior stroke, or heart failure) [45]. In instances where episodes of atrial fibrillation lasting ≥6 min were detected, anticoagulation was initiated. Interestingly, the authors found no correlation between implantable loop recorder-detected atrial fibrillation and/or subclinical atrial fibrillation burden with annual Montreal Cognitive Assessment scores over a three-year follow-up period [44]. The authors hypothesised that the study’s negative results may have been attributable to the early detection and treatment of atrial fibrillation with anticoagulant therapy. However, it is worth noting that subjects were excluded where annual Montreal Cognitive Assessment score data were unavailable, and this may have confounded the study’s results.

The observational nature of the studies to date makes it difficult to make firm conclusions about the effects of anticoagulant therapy use on the risk of dementia. Prospective studies are warranted. The Blinded Randomised Trial of Anticoagulation to Prevent Ischaemic Stroke and Neurocognitive Impairment in Atrial Fibrillation (BRAIN-AF) study (ClinicalTrials.gov #NCT02387229) will randomise patients to either rivaroxaban 15 mg once daily or standard of care, and examine cognitive outcomes in atrial fibrillation patients aged 30–62 with a low risk of stroke. The study will aim to ascertain whether initiating anticoagulant therapy earlier in the atrial fibrillation disease course is of benefit in the prevention of dementia. In such circumstances, the potential benefits of anticoagulant therapy will need to be carefully balanced against a possible increased bleeding risk.

Several studies have attempted to determine whether the type of anticoagulant used (vitamin K antagonist versus direct-acting oral anticoagulant) affects dementia risk in atrial fibrillation. Observational studies have shown that the risk of cognitive impairment in patients taking direct-acting oral anticoagulants may be reduced [46,47], unaffected [41], or possibly even increased in patients ≥80 years [48], when compared to vitamin K antagonists (Table 1). As highlighted in Table 1, these cohort studies used a diagnosis of dementia as their outcome measure, and it is unclear exactly how the diagnoses were reached. Decision making regarding choice of anticoagulant therapy may also have been influenced by unmeasured confounders. Studies of this nature are additionally unable to accurately determine medication compliance rates.

Prospective randomised trials have subsequently been performed to examine cognitive outcomes in patients taking dabigatran versus dose-adjusted vitamin K antagonist (Table 2) [49,50]. The Cognitive Decline and Dementia in Patients with Nonvalvular Atrial Fibrillation (CAF) and The Cognitive Impairment Related to Atrial Fibrillation Prevention (GIRAF) trials both failed to demonstrate a difference in cognitive outcomes amongst patients treated with warfarin or dabigatran. As highlighted in Table 2, these studies used a variety of different cognitive function assessments to examine the potential link between the type of anticoagulant therapy used and cognition.

### 3.2. Left Atrial Appendage Occlusion

In patients deemed unsuitable for anticoagulation therapy, left atrial appendage occlusion has emerged as a potential treatment option to reduce the risk of systemic thromboembolism including stroke [51]. Whilst oral anticoagulants may reduce the risk of cerebral emboli developing, their use has been associated with the development of cerebral microbleeds which may be associated with worsening cognitive performance [15]. It has been postulated that left atrial appendage occlusion may reduce the incidence of cerebral emboli and concomitantly result in fewer cerebral microbleeds. However, the procedure has also been associated with a risk of periprocedural stroke [52]. The development of cerebral infarcts in these instances may result in cognitive decline. It is therefore unclear how left atrial appendage occlusion may modify the risk of atrial fibrillation-associated cognitive decline.

In a study by Mohanty et al., the cognitive effects of anticoagulant therapy versus left atrial appendage occlusion were examined in a 1-year follow-up of 98 patients after catheter ablation [53]. The use of anticoagulant therapy was associated with a significant decline in Montreal Cognitive Assessment scores following ablation. Interestingly, this was not observed with left atrial appendage occlusion. It is important to note that the study was non-randomised and should therefore be considered exploratory. A selection bias favouring the use of left atrial appendage occlusion in healthier patients less likely to develop cognitive impairment presents a possible explanation for the study’s results. It may be reasonable to surmise that patients deemed unsuitable for left atrial appendage occlusion may have had a higher baseline risk of cognitive impairment.

The PLUG Dementia trial and its MRI PLUG dementia sub-study (ClinicalTrials.gov #NCT03091855) were designed to further ascertain the cognitive effects of left atrial appendage occlusion therapy. Unfortunately, the trial and its sub-study were terminated due to low enrolment. At present there are insufficient data to accurately determine the impact of left atrial appendage occlusion on cognition in atrial fibrillation. Future studies should be designed to examine the effects of the treatment on cognition, as well as the incidence of cerebral emboli and microbleeds.

## 4. Rhythm Control

The achievement of sinus rhythm with rhythm control therapy may result in improved cardiac output and cerebral perfusion offering the potential to mitigate the risk of atrial fibrillation-related cognitive decline. This theory is supported by the observation that cerebral perfusion is reduced in patients with atrial fibrillation compared with those in normal sinus rhythm [30]. Currently it is unclear whether these changes in cerebral perfusion patterns affect cognitive outcomes. Efforts to restore sinus rhythm and subsequently improve cerebral perfusion have been hypothesised to improve cognitive outcomes in patients with atrial fibrillation. However, it is well recognised that procedures such as cardioversion and catheter ablation confer an increased risk of periprocedural stroke [54,55]. It is consequently feasible to speculate that rhythm control therapy could cause cerebral emboli which could adversely affect cognitive outcomes. Studies have therefore been performed to attempt to determine the impact of catheter ablation [56,57,58,59,60,61,62,63], cardioversion [64,65,66,67,68], and pharmacological rhythm control therapy [69,70,71] on atrial fibrillation-associated cognitive decline.

### 4.1. Catheter Ablation

The effects of catheter ablation on cognitive function in atrial fibrillation are unknown and have been reviewed previously by our research group [72]. The restoration of sinus rhythm with catheter ablation has been demonstrated to result in improved cerebral perfusion [73,74,75]. However, catheter ablation has also been associated with an increased risk of asymptomatic cerebral emboli, particularly in the immediate post-ablation period [76]. Several studies have been performed to ascertain the effects of catheter ablation on cognitive function [56,57,58,59,60,61,62,63]. Whilst observational data indicates a possible association between the procedure and a reduced risk of cognitive impairment, there is a significant lack of randomised controlled trial evidence to make any firm conclusions about the effects of catheter ablation on cognitive function [72].

Prospective trials are underway and aiming to compare the impact of catheter ablation versus medical therapy on cognitive function. The Cognitive Impairment in Atrial Fibrillation study (DIAL-F) (ClinicalTrials.gov NCT01816308) will aim to compare cognition in patients treated with catheter ablation versus anti-arrhythmic drug therapy, in an observational case-control study comprising 888 participants aged 50–75, over a 2-year follow-up period. The Neurocognition and Greater Maintenance of Sinus Rhythm in Atrial Fibrillation (NOGGIN AF, project number 1R01AG07418501) trial has also recently been announced. Whilst further details of this prospective, observational clinical trial are currently awaited, it is known that the study will aim to compare cognitive function, structural cortical characteristics, and cerebral blood flow in patients treated with catheter ablation versus those treated with medical therapy.

### 4.2. Cardioversion

The impact of electrical cardioversion on cognitive function remains unclear. In patients who undergo the procedure electively, it has been demonstrated that restoration and maintenance of sinus rhythm after at least ten weeks is associated with magnetic resonance imaging-detected improvements in cerebral perfusion and blood flow [68]. Interestingly, these improvements were not observed where electrical cardioversion was unsuccessful. Whether or not these changes in cerebral perfusion patterns translate to a long-term improvement in cognitive function remains an area for further study, although given the high rate of recurrence of atrial fibrillation following cardioversion [77] a long-term improvement in cognition may be unlikely.

Studies have also been performed to determine the incidence of cerebral emboli following cardioversion (Table 3) [64,65,66,67]. Bernhardt et al., observed an incidence of 4.7% in their study, whilst studies by Vázquez et al., Bellman et al., and Arvanitis et al., did not show any new cerebral emboli post-cardioversion. As highlighted in Table 3, these studies varied in terms of the patient populations studied, methods of anticoagulation, and timing of cerebral magnetic resonance imaging scans post-cardioversion, and this may account for the discrepant findings.

In the future, studies should attempt to ascertain the longer-term effects of cardioversions on neurocognitive function, and delineate whether certain patient groups are at a higher risk of cerebral emboli and potential cognitive impairment. The NOR-FIB2 study (Fibrosis, Inflammation and Brain Health in Atrial Fibrillation: The Norwegian Atrial Fibrillation and Stroke Study) (ClinicalTrials.gov #NCT03816865) will attempt to assess the impact of inflammation (measured by biomarkers and cardiac 18F-fluorodeoxyglucose-positron emission tomography) on the risk of new silent cerebral lesions following electrical cardioversion. This study may explain the variation in observed incidence of cerebral emboli amongst the studies listed in Table 3.

### 4.3. Pharmacological Rhythm Control Therapy

Studies which have examined the effects of pharmacological rhythm control strategies on cognitive function have yielded conflicting results. When using a combination of anti-arrhythmic drugs and ablation for rhythm control, the Early Treatment of Atrial Fibrillation for Stroke Prevention trial (EAST-AFNET 4) [69] failed to show a significant difference in change in cognitive function assessed by the Montreal Cognitive Assessment at two years between patients treated with early rhythm control therapy versus usual care. This study corroborated the findings of a sub-study of the Atrial Fibrillation Follow-up Investigation of Rhythm Management (AFFIRM) trial, which found no differences in mini mental state examination scores in patients treated with rhythm control therapy versus rate control therapy [78].

However, it is important to highlight that cognitive function assessment scores were secondary outcomes in these studies, and that they may therefore have been underpowered to detect differences in cognitive function. Furthermore, it has been argued that the follow-up periods for these studies may have been too short. In a study by Lin et al., atrial fibrillation patients receiving anti-arrhythmic drugs were found to have lower rates of dementia compared with patients receiving rate control therapy over a 4.86 ± 3.38-year follow-up period [71]. This finding confirmed the results of a study by Damanti et al., which assessed cognitive performance using the Short Blessed Test in patients treated with rhythm and rate control therapy. The authors found that cognitive performance was higher in the rhythm control therapy group [70].

Given the variations in study design and outcome measures of the studies performed to date, it is currently difficult to comment on the impact of pharmacological rhythm control therapy on the risk of atrial fibrillation-associated cognitive decline. Further studies should attempt to evaluate the impact of pharmacological rhythm control strategies on the putative mechanisms linking atrial fibrillation and cognitive decline. The impact of pharmacological rhythm control on cerebral perfusion patterns and the incidence of cerebral emboli is currently unknown, and presents an important area for further study. 

Given the variable efficacy of anti-arrhythmic drugs in maintaining sinus rhythm, studies should also be designed to measure atrial fibrillation burden and examine whether it correlates with cognitive outcomes. Tang et al., have recently identified a potential link between atrial fibrillation burden and cognitive function in a prospective cohort study of 253 patients with established diagnoses of atrial fibrillation [79]. The authors found that patients with higher burdens of atrial fibrillation appeared to have lower Montreal Cognitive Assessment scores. Whilst this study demonstrates a potential association between atrial fibrillation burden and cognitive function, it is unable to determine how a reduction in atrial fibrillation burden may affect cognitive outcomes. Future studies should aim to decipher the precise link between atrial fibrillation burden and cognitive function, and examine whether a reduction in atrial fibrillation burden has an impact on the progression of atrial fibrillation-associated cognitive decline.

## 5. Rate Control

The optimal rate control strategy to mitigate the risk of atrial fibrillation-associated cognitive decline is unclear. Interestingly, a ten-year cohort study by Cacciatore et al., found that the ventricular rate response during atrial fibrillation may be associated with an increased risk of dementia [80]. Patients were stratified into groups with a low/high (<50/>90 beats per minute) and moderate (>50/<90 beats per minute) ventricular rate response during atrial fibrillation. The authors found that the risk of dementia was heightened in patients in the low/high ventricular rate response group (hazard ratio 7.70, 95% confidence interval 1.10–14.20, *p* = 0.03). The study assessed rate response using a 24-h Holter monitor, and it is unclear when exactly this investigation was performed on study participants. We therefore do not have a precise estimate of ventricular rate control during the follow-up period. It is also noteworthy that the confidence intervals were relatively large, and it is therefore difficult to make any firm conclusions based on the results of this study.

It has been proposed that the ventricular response rate may affect cerebral perfusion patterns in atrial fibrillation. This was investigated in a study by Saglietto et al., where computational modelling was utilised to investigate whether ventricular response rate during atrial fibrillation may impact cerebral haemodynamics [81]. The authors found that higher ventricular rates related to a progressive increase in critical cerebral haemodynamic events (hypoperfusions and hypertensive events) at the distal cerebral circle (downstream from the middle cerebral artery). Their data suggested that a rate control strategy aiming for around 60 beats per minute may be beneficial for cerebral perfusion and cognitive outcomes in permanent atrial fibrillation. The current European Society of Cardiology guidelines state that a heart rate < 110 beats per minute should be targeted in atrial fibrillation [25]. This evidence is based on the Lenient versus Strict Rate Control in Patients with Atrial Fibrillation (RACE II) trial, which found no significant differences in outcomes amongst the lenient and strict rate control arms of the study [82]. However, this study did not examine cognitive outcomes. Further studies are therefore warranted to determine whether there is an optimal target heart rate to mitigate the risk of atrial fibrillation-associated cognitive decline.

It has been postulated that atrial fibrillation-induced RR interval variability may adversely affect cerebral blood flow [30,83]. Therefore, regularisation of the RR interval may lead to improvements in cerebral perfusion. To support this mechanism, it has been demonstrated that the use of a ‘pace and ablate’ strategy in patients with medically refractory rapidly conducted atrial fibrillation has been associated with improved cerebral blood flow measured by brain single photon emission computed tomography scanning with (99 m) Tc-hexamethylpropylene amine oxime and improved cognitive performance at 3 months [84]. These findings warrant further investigation to ascertain whether the use of a pacemaker, which could correct atrial fibrillation-induced RR variability, may positively affect cerebral perfusion patterns and subsequently reduce the risk of cognitive decline.

## 6. Risk Factor Management

Atrial fibrillation and dementia share risk factors which may contribute to the link between the two disease states. Heart failure, diabetes, hypertension, smoking, physical inactivity, and obstructive sleep apnoea have been identified as risk factors for both atrial fibrillation and cognitive decline [85]. However, studies which have controlled for these risk factors have demonstrated that atrial fibrillation itself remains an independent risk factor for cognitive decline [12,13]. Whilst atrial fibrillation itself is thought to be independently associated with cognitive impairment, risk factor management is likely to play a significant role in mitigating the risk of atrial fibrillation-associated dementia.

### 6.1. Heart Failure

Evidence is accumulating to suggest that the incidence of dementia may be higher in heart failure patients [86], and several candidate pathophysiological mechanisms have subsequently been proposed to explain this association [87,88,89]. The observation that cerebral blood flow may be reduced by up to 30% in severe heart failure has resulted in speculation that heart failure-induced cerebral hypoperfusion may cause cognitive decline [87]. Furthermore, it has previously been shown that heart failure patients have an increased prevalence of cerebral cortical microinfarcts [88], thus presenting another putative mechanism linking heart failure and cognitive impairment. Cerebral inflammation has been proposed as another candidate pathophysiological mechanism linking heart failure and cognitive decline [90]. Studies have shown that patients with heart failure with reduced ejection fraction have elevated levels of pro-inflammatory cytokines compared with healthy individuals [89], and it has also been demonstrated that blood-brain barrier permeability is increased in rat models of ischaemic heart failure [91]. This increased permeability may therefore facilitate the entry of pro-inflammatory cytokines leading to cognitive decline. Cerebral hypoperfusion [68,87], cerebral infarction [19,88], and systemic inflammation [37,89] consequently represent common candidate pathophysiological mechanisms linking atrial fibrillation, heart failure, and cognitive decline. Further studies are warranted to investigate these mechanistic links and to ascertain the impact of a combined diagnosis of the conditions compared with either risk factor alone on cognitive function. This is particularly important in the atrial fibrillation population, since the condition is associated with a markedly increased heart failure risk [92].

Heart failure therapies such as cardiac resynchronisation therapy have previously been shown to result in improved cerebral perfusion patterns in patients with heart failure [93]. Future studies should aim to explore the impacts of such treatment modalities on cerebral perfusion patterns and cognitive function in patients with atrial fibrillation and heart failure. Given the hypothesis that cerebral infarction may be a common candidate pathophysiological mechanism linking atrial fibrillation, heart failure, and cognitive decline, it appears reasonable to hypothesise that anticoagulation therapy may reduce the risk of cognitive impairment in patients with atrial fibrillation and heart failure. Amongst patients with systolic heart failure in sinus rhythm, a post-hoc analysis of the Warfarin versus Aspirin in Reduced Cardiac Ejection Fraction (WARCEF) trial failed to demonstrate an association between cognitive decline and the use of warfarin or aspirin [94]. The impact of anticoagulant therapy on cognitive function in patients with atrial fibrillation and heart failure is currently unclear, and studies should be prospectively designed and adequately powered to assess the effects of anticoagulation therapy on cognitive outcomes in patients with atrial fibrillation and heart failure.

### 6.2. Diabetes

Diabetes mellitus has been associated with an increased risk of dementia [95,96]. Previously, it has been hypothesised that diabetes mellitus-induced hyperglycaemia may cause inflammation and oxidative stress resulting in brain insulin resistance and subsequent neurodegeneration [97]. Inflammation therefore presents a common candidate pathophysiological mechanism linking atrial fibrillation, diabetes mellitus, and cognitive decline. Observational studies have demonstrated that amongst patients with an atrial fibrillation diagnosis, patients with diabetes mellitus appear to have higher rates of dementia compared with those without diabetes mellitus [98]. It is important to note that such studies cannot infer a causal link between either diabetes mellitus and/or atrial fibrillation with dementia. Nonetheless, they should be considered hypothesis-generating, and further studies should examine exactly how atrial fibrillation and diabetes may interact to affect the trajectory of atrial fibrillation-associated cognitive impairment.

### 6.3. Blood Pressure Management

The optimal blood pressure target in atrial fibrillation is unknown. A post-hoc analysis of the Systolic Blood Pressure Intervention (SPRINT) trial investigated whether a diagnosis of atrial fibrillation affected the treatment effects of intensive blood pressure control (target systolic blood pressure < 120 mmHg) versus standard blood pressure control (target systolic blood pressure < 140 mmHg) [99]. Interestingly, in atrial fibrillation patients, intensive blood pressure control was associated with a lower risk of cardiovascular events but a higher risk of dementia. It is important to note that these results were obtained from a post-hoc analysis and should therefore be considered exploratory. However, since cerebral hypoperfusion is a purported mechanism by which atrial fibrillation may cause cognitive decline, it appears feasible to speculate that the optimal blood pressure target to prevent cognitive decline in atrial fibrillation may be different to that for the general population. In patients without a diagnosis of atrial fibrillation, a U-shaped association between blood pressure and cognitive function has been observed [100]. It is unknown whether such a pattern persists for the atrial fibrillation population.

### 6.4. Smoking

Smoking is recognised as a shared risk factor for atrial fibrillation and dementia. However, the importance of smoking cessation is not always heavily asserted in the management of atrial fibrillation [3]. Lee et al., observed that smoking cessation after a diagnosis of atrial fibrillation may be associated with a lower dementia risk in a cohort study of 126,252 atrial fibrillation patients from the Korean National Health Insurance Service database [101]. Given the nature of the study design however, we can only infer an association as opposed to causation. Nevertheless, the study provides support to emphasise the importance of smoking cessation in the management of atrial fibrillation. Future studies should aim to confirm the findings of the study by Lee et al., and delineate whether smoking in combination with atrial fibrillation confers a superimposed cognitive impairment risk compared with either risk factor alone. This may lead to the adoption of more rigorous efforts to promote smoking cessation in atrial fibrillation patients.

### 6.5. Physical Activity

In the general population, physical activity has been suggested as a treatment strategy by the World Health Organisation, to reduce the risk of dementia [102]. In patients with newly diagnosed atrial fibrillation, Lim et al., showed that patients who initiated regular exercise had a lower risk of dementia in comparison to non-exercisers (hazard ratio 0.87, 95% confidence interval 0.81–0.94) in a nationwide cohort study of 126,555 patients with atrial fibrillation [103]. Given the study’s observational nature, causation cannot be inferred. Nonetheless, the findings warrant further investigation. It would be prudent for studies which examine the impact of physical activity and cardiac rehabilitation programmes in atrial fibrillation to include cognitive outcomes as endpoints. Obesity is also well-recognised as a risk factor for dementia [104] and atrial fibrillation [105]. Studies should also attempt to ascertain whether physical activity could potentially lessen the risk of atrial fibrillation-associated cognitive decline through weight reduction or potential alternate mechanisms.

### 6.6. Obstructive Sleep Apnoea

A high prevalence of obstructive sleep apnoea has been observed in patients with atrial fibrillation [106]. The precise mechanistic link between the two disease states is currently unclear and under further investigation [107]. Obstructive sleep apnoea has also been identified as an independent risk factor for dementia [108]. To our knowledge, no studies have been performed to ascertain the effects that a combination of obstructive sleep apnoea and atrial fibrillation may have on cognitive outcomes. Studies which aim to explore the link between obstructive sleep apnoea and atrial fibrillation should therefore also investigate the combined cognitive effects of these conditions. In the future, targeted treatment strategies may potentially be able to halt the development of atrial fibrillation-associated as well as obstructive sleep apnoea-associated cognitive decline.

## 7. Areas for Further Study

This review has highlighted multiple unanswered questions in the management of atrial fibrillation and cognitive impairment (summarised in Table 4). It is currently unclear whether atrial fibrillation-associated cognitive decline should be considered as a contributing factor to other forms of dementia, or as a separate disease process entirely. Future studies should aim to determine whether atrial fibrillation-associated cognitive decline differs from other forms of dementia and to ascertain whether certain cognitive domains are affected to a greater extent than others in patients with atrial fibrillation. Studies should also be designed to determine whether there is an optimal cognitive screening assessment tool to detect the early stages of atrial fibrillation-associated cognitive decline.

Social determinants, such as ethnicity, access to health care, and social support, are increasingly becoming recognised as important contributors to outcomes amongst patients with atrial fibrillation [109]. Amongst the general population, studies have shown that health and socioeconomic disparities appear to be stronger determinants of dementia prevalence than race or cultural identifiers [110]. Future studies should consequently aim to ascertain the impact of social determinants of health on cognitive outcomes amongst patients with atrial fibrillation, and identify the precise social determinants of health that need to be targeted in order to reduce patients’ risk of atrial fibrillation-associated cognitive decline.

Studies which attempt to examine the link between atrial fibrillation and cognitive impairment will need to be carefully designed to ensure that the relationship between atrial fibrillation and dementia is fully understood. By definition, studies will require lengthy follow-up periods, and this introduces the potential for both semi-competing events (e.g., death) and competing events (e.g., ischaemic stroke) to mask potential associations [111]. Furthermore, patients who have or who are at risk of dementia often tend to have multiple comorbidities. Indeed, it is well recognised that frailty and cognitive impairment are closely related in the general population [112]. Studies should aim to examine the relationship between frailty and cognitive impairment amongst atrial fibrillation patients. Historically, older patients with multiple comorbidities have been underrepresented in research studies [113]. Efforts should consequently be made to ensure that studies exploring the link between atrial fibrillation and cognitive impairment include the full spectrum of atrial fibrillation patients, to ensure that findings accurately ascertain the relationship between atrial fibrillation and cognitive impairment.

## 8. Conclusions

There is a growing evidence base to suggest an association between atrial fibrillation and cognitive impairment. The mechanistic link between the two disease states is currently unclear, thus warranting further study. Existing treatment modalities for atrial fibrillation may be able to influence the cognitive decline process. However, the current evidence base is largely observational, asserting the requirement for randomised controlled trial evidence. Furthermore, controversy regarding the relationship between atrial fibrillation and cognition may result from differences in definitions, methodologies, and assessment techniques for cognitive function. Future studies investigating atrial fibrillation treatment strategies should incorporate outcomes sensitive to the putative cognitive consequences of atrial fibrillation.

## Figures and Tables

**Table 1:** Studies which examine the impact of direct oral anticoagulant therapy versus vitamin K antagonist therapy on cognitive outcomes.

Title	Total Number of Patients Receiving Oral Anticoagulant Therapy in the Study	Number of Patients Treated with Novel Oral Anticoagulants	Number of Patients Treated with Vitamin K Antagonist	Major Study Findings
Association of Oral Anticoagulant Type With Risk of Dementia Among Patients With Nonvalvular Atrial Fibrillation [46]	468,601	139,463	329,138	The patients treated with direct oral anticoagulants had lower rates of dementia than patients treated with vitamin K antagonists (dabigatran: hazard ratio 0.85 (95% confidence interval 0.71–1.01); rivaroxaban: hazard ratio 0.85 (95% confidence interval 0.76–0.94); apixaban: hazard ratio 0.80 (95% confidence interval 0.65–0.97)).
Less Dementia and Stroke in Low-Risk Patients with Atrial Fibrillation Taking Oral Anticoagulation [41]	39,160	2926	36,234	The primary outcome measure was a composite of stroke, dementia, and intracranial haemorrhage. The hazard ratio for direct oral anticoagulants versus vitamin K antagonist was 0.47 (95% confidence interval 0.18–1.22).
Nonvitamin K Antagonist Oral Anticoagulants Versus Warfarin in Atrial Fibrillation Patients and Risk of Dementia: A Nationwide Propensity-Weighted Cohort Study [48]	34,683	21,311	13,372	Rates of dementia appeared to vary according to age group, with no significant differences in patients <80. In patients ≥80, direct oral anticoagulant use was associated with a higher risk of dementia (hazard ratio 1.31 (95% confidence interval 1.07–1.59)).
Oral Anticoagulant Treatment and the Risk of Dementia in Patients With Atrial Fibrillation: A Population-Based Cohort Study [47]	11,419 A proportion of the patients were treated with both novel oral anticoagulants and vitamin K antagonists during the study (n = 3176).	5570	2673	Patients receiving direct oral anticoagulants had a lower incidence of dementia than patients treated with vitamin K antagonists (hazard ratio 0.46 (95% confidence interval 0.28–0.74)).

**Table 2:** Studies which have compared the impact of warfarin versus dabigatran on cognitive function in patients with atrial fibrillation.

Study Name	Number of Participants	Warfarin Details	Dabigatran Details	Outcome Measures	Timeframe	Major Study Findings
Impact of Anticoagulation Therapy on the Cognitive Decline and Dementia in Patients with Non-Valvular Atrial Fibrillation (Cognitive Decline and Dementia in Patients with Non-Valvular Atrial Fibrillation [CAF] trial) [49]	101	Warfarin once daily. INR target 2–3.	Dabigatran (150 mg twice daily if creatinine clearance > 30 mL/min, or 75 mg twice daily if CrCL > 15 to 30 mL/min or dose as per United States package insert)	(1) Formal diagnosis of dementia by a neurologist; (2) Moderate decline in cognitive function based on the Alzheimer’s Disease Assessment Scale and the Disability Assessment for Dementia	24 months	The use of dabigatran and warfarin therapy were associated with similar risks of cognitive decline at two years.
Effects of Dabigatran versus Warfarin on 2-year Cognitive Outcomes in Old Patients with Atrial Fibrillation: Results from the GIRAF Randomized Clinical Trial [50]	200	Warfarin once daily. INR target 2–3.	Dabigatran 150 mg twice daily	Changes in the following cognitive assessment scores at two years: Mini mental state examination; A composite neurological psychological test battery (NTB); Computer generated tests (CGNT); Montreal cognitive assessment score	24 months	Cognitive outcomes did not differ amongst patients treated with dabigatran or warfarin (time in therapeutic range ≥70%).

**Table 3:** Summary of studies which have examined the impact of electrical cardioversion on incidence of asymptomatic cerebral emboli.

Title	Total Number of Patients Included in the Study	Observed Incidence of Cerebral Emboli (n (%))	Timing of Cerebral Magnetic Resonance Imaging	Anticoagulation
Incidence of Cerebral Embolism after Cardioversion of Atrial Fibrillation: A Prospective Study with Transesophageal Echocardiography and Cerebral Magnetic Resonance Imaging [64]	127	6/127 (4.7%)	Magnetic resonance imaging scans performed serially over a period of four weeks.	All patients received vitamin K antagonist therapy for four weeks before and four weeks after cardioversion.
Incidence of MRI-Detected Brain Lesions and Neurocognitive Function after Electrical Cardioversion in Anticoagulated Patients with Persistent Atrial Fibrillation [65]	50	0/50 (0%)	Performed 24 h before and 2 weeks after cardioversion	A total of 39 patients were treated with direct oral anticoagulant (Dabigatran 10/50 [20%], Apixaban 21/50 [42%], and Rivaroxaban 8/50 [16]) and 11/50 patients with Phenprocoumon (22%).
Assessment of Silent Microembolism by Magnetic Resonance Imaging After Cardioversion in Atrial Fibrillation [66]	62	0/62 (0%)	Magnetic resonance imaging performed immediately before and 24 h after cardioversion	All patients received acenocoumarol to maintain an international normalised ratio between 2 and 3.
Serial Magnetic Resonance Imaging after Electrical Cardioversion of Recent Onset Atrial Fibrillation in Anticoagulant-Naïve Patients—A Prospective Study Exploring Clinically Silent Cerebral Lesions [67]	43	0/43 (0%)	Magnetic resonance imaging scan performed at baseline (pre cardioversion), immediately post-cardioversion, and 7–10 days after cardioversion.	This study examined patients with recent onset atrial fibrillation. Therefore, patients were anticoagulant naïve. After cardioversion, 8/43 (18.6%) patients received treatment with novel oral anticoagulants.

**Table 4:** Summary of unanswered research questions highlighted within the article.

Unanswered Research Questions in the Management of Atrial Fibrillation-Associated Cognitive Impairment
Is there a way of distinguishing atrial fibrillation-associated cognitive decline from other forms of dementia?
How does use of the ‘Atrial Fibrillation Better Care’ pathway modify the risk of atrial fibrillation-associated cognitive decline?
How do social determinants of health affect a patient’s risk of atrial fibrillation-associated cognitive impairment?
**Mechanisms of atrial fibrillation-associated cognitive decline**
What is the mechanism of interaction between silent cerebral infarcts and cognitive decline?
How are atrial cardiomyopathy and cognitive decline linked?
What is the impact of atrial fibrillation-associated cerebral hypoperfusion on cognitive outcomes?How do cerebral microbleeds develop in patients with atrial fibrillation?
Do cerebral microbleeds contribute to atrial fibrillation-associated cognitive decline?
What role does inflammation play in the pathophysiology of atrial fibrillation-associated cognitive decline?
**Stroke risk reduction**
What is the impact of oral anticoagulation in atrial fibrillation patients deemed to be at lower risk of systemic thromboembolism?
Does early commencement of anticoagulant therapy mitigate the risk of atrial fibrillation-associated cognitive decline?
How does left atrial appendage occlusion modify the risk of atrial fibrillation-associated cognitive decline?
**Rhythm control**
What is the impact of catheter ablation versus medical therapy on cognitive outcomes?
What effect does electrical cardioversion have on cognitive function?
How does pharmacological rhythm control therapy affect cognitive outcomes?
Can a reduction in atrial fibrillation burden slow the progression of atrial fibrillation-associated cognitive decline?
**Rate control**
Is there an optimal rate control strategy to prevent atrial fibrillation-associated cognitive decline?
What are the effects of ‘pace and ablate’ versus rate control therapy on cognitive outcomes?How do variations in RR interval variability during atrial fibrillation affect cerebral perfusion patterns and the risk of cognitive impairment?
**Risk factor management**
How does a combined diagnosis of atrial fibrillation and heart failure affect the risk of cognitive impairment?
Do atrial fibrillation patients with a diagnosis of diabetes mellitus have an increased risk of cognitive decline?
Is there an optimal blood pressure target to mitigate the risk of atrial fibrillation-associated cognitive decline?
Does smoking confer a superimposed risk of cognitive impairment in atrial fibrillation?
Can physical activity prevent atrial fibrillation-associated cognitive decline?
How does obstructive sleep apnoea affect atrial fibrillation-associated cognitive decline?

## Data Availability

Not applicable.
